# Fractures sus et inter-condyliennes de l’humérus distal chez l’adulte

**DOI:** 10.11604/pamj.2020.36.346.24516

**Published:** 2020-08-25

**Authors:** Meryem Lemsanni, Rachid Chafik, Mohamed Madhar, Hanane Elhaoury, Youssef Najeb

**Affiliations:** 1 Service de Chirurgie Orthopédique et Traumatologique, Hôpital Ibn Tofail, Centre Hospitalier Universitaire Mohammed VI, Marrakech, Maroc

**Keywords:** Fracture, humérus distal, articulaire, sus et inter-condylienne, ostéosynthèse, abord postérieur, trans-olécranien, Fracture, distal humerus, articular, sub and intercondylian, osteosynthesis, posterior approach, transolecranon

## Abstract

**Introduction:**

les fractures articulaires complètes de l’extrémité inférieure de l’humérus de l’adulte sont des lésions rares et graves. Les options thérapeutiques sont nombreuses mais le traitement chirurgical par ostéosynthèse est ardemment défendu. L’objectif de notre travail a été de décrire les caractéristiques épidémiologiques, clinico-radiologiques et thérapeutiques de ces fractures, ainsi que d’évaluer les résultats fonctionnels obtenus chez nos patients.

**Méthodes:**

nous avons mené une étude prospective sur une période de 3 ans, portant sur 38 patients admis pour fracture articulaire complète sus et inter-condylienne de l’humérus distal (classée type C selon la classification de l’AO), traités chirurgicalement par voie postérieure trans-olécranienne avec un recul moyen de 34 mois.

**Résultats:**

nous avons remarqué une distribution bimodale avec une atteinte du sujet jeune de sexe masculin d’une part, et une survenue chez les femmes âgées d’autre part. Les étiologies étaient dominées par les accidents de la voie publique chez 78%. Lors du suivi, nous avons noté un seul cas d’infection superficielle du site opératoire et il n’y a eu aucun cas de démontage du matériel ni de pseudarthrose. De surcroit, aucune complication de l’ostéosynthèse de l’olécrane n’a été enregistrée. Les résultats fonctionnels ont été très satisfaisants avec un score de Mayo-Clinic Elbow Performance Score (MEPS) moyen de 86.

**Conclusion:**

nous considérons que la voie postérieure trans-olécranienne semble être la meilleure voie d’abord de ces fractures puisqu’elle permet une bonne exposition articulaire, condition sine qua non pour une restitution anatomique parfaite et une ostéosynthèse stable afin d’entreprendre une rééducation précoce et adaptée.

## Abstract (en)

**Introduction:**

complete joint fractures of the lower end of the humerus are rare and severe injuries in adults, affecting the functional prognosis of a narrow and complex joint such as the elbow. Multiple therapeutic options are available, but osteosynthesis is widely used provided that it can recover stable, mobile, and painless joint. The purpose of our study was to describe the epidemiological, clinical, radiological and therapeutic features of these fractures, as well as to evaluate functional outcomes in our patients.

**Methods:**

we conducted a 3-year prospective study (January 2014-December 2016) of 38 patients older than 16 years admitted with complete sub- and intercondylar joint fracture of the lower extremity of the humerus (classified as type C according to the AO classification). They underwent surgery via transolecranian posterior approach with fixation of one or two screw plates based on anatomopathological diagnosis. Patients were followed-up for a mean period of 34 months.

**Results:**

these conditions had bimodal distribution, affecting young male subjects on the one hand and elderly female subjects on the other. The main cause of fractures was road accidents (78%). During the follow-up period, one case had superficial surgical site infection and there were no cases of dismantling of equipment or pseudarthrosis. No complications after osteosynthesis for olecranon fractures were recorded in our case series. Stiffness at the elbow-joint was defined as bending/extension arc less than 50°; elbow stiffness was found in 25% of patients. Functional results were assessed using the Mayo-Clinic or Mayo-Clinic Elbow Performance Score (MEPS) with an average score of 86.

**Conclusion:**

based on our results, transolecranon posterior approach is the best option for these fractures, since it allows good joint exposure. This is essential for perfect anatomical restitution and stable osteosynthesis in order to undertake early and adapted rehabilitation.

10 Feb 2021: Corrigendum: Fractures sus et inter-condyliennes de l’humérus distal chez l’adulte. Pan African Medical Journal. 2021.38.152. doi: 10.11604/pamj.2021.38.152.28075

## Introduction

Les fractures articulaires complètes de l’extrémité inférieure de l’humérus (EIH) de l’adulte sont des lésions rares et graves puisqu’elles engagent le pronostic fonctionnel d’une articulation étroite et complexe qu’est le coude. Les options thérapeutiques sont nombreuses et vont de l’immobilisation orthopédique à l’arthroplastie totale du coude en passant par le traitement chirurgical qui occupe une place très importante dans la prise en charge de ce type de fractures. Ce traitement, malgré une multitude de voies d’abord et de techniques chirurgicales, doit répondre au cahier des charges standard de toute fracture articulaire et notamment la récupération d’une articulation mobile, stable et indolore. L’objectif de notre travail a été de décrire les caractéristiques épidémiologiques, clinico-radiologiques et thérapeutiques de ces fractures, ainsi que d’évaluer les résultats fonctionnels obtenus chez nos patients.

## Méthodes

Nous avons mené une étude prospective sur une période de 3 ans (janvier 2014-décembre 2016), portant sur 38 patients âgés de plus de 16 ans, admis pour fracture articulaire complète sus et inter-condylienne de l’EIH (classée type C selon la classification de l’AO), traités chirurgicalement par voie postérieure trans-olécranienne avec mise en place d’une ou deux plaques vissées en fonction du type anatomo-pathologique. Les paramètres étudiés étaient: l’âge, le sexe, les circonstances traumatiques, les lésions associées, les signes cliniques, les résultats du bilan radiologique, le délai opératoire, le type d’anesthésie, la technique chirurgicale (installation, voie d’abord, nature du matériel d’ostéosynthèse), les soins post-opératoires, l’évolution (favorable ou compliquée), le suivi radiologique (consolidation normale ou pathologique). Ces données ont été collectées à partir des dossiers médicaux. Les résultats fonctionnels ont été évalués selon le score de performance du coude de la Mayo-Clinic ou MEPS. Les patients ont été revus après 3 semaines, 6 semaines, 3 mois, 6 mois puis annuellement. Le recul moyen a été de 34 mois. L’analyse statistique était réalisée à l’aide du logiciel SPSS version 17.

## Résultats

**Figure 1:**
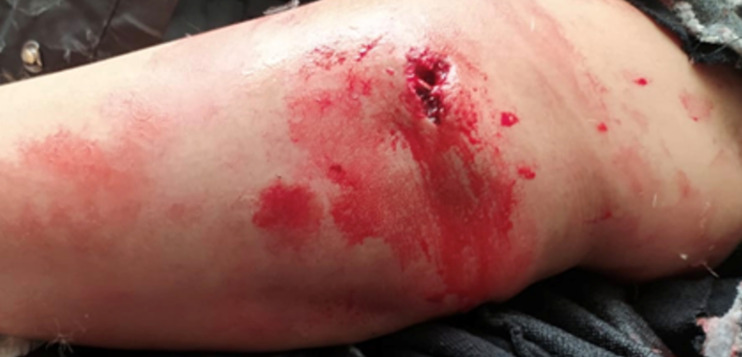
plaie punctiforme en regard du capitellum

**Figure 2:**
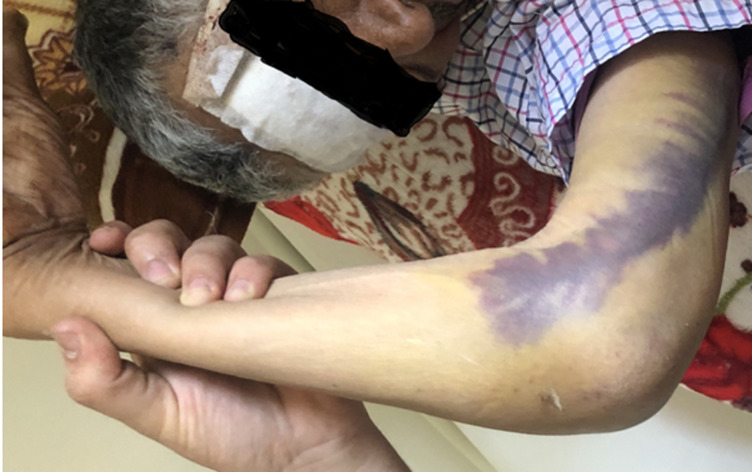
large ecchymose s’étendant le long de la face interne du bras et du coude

**Figure 3:**
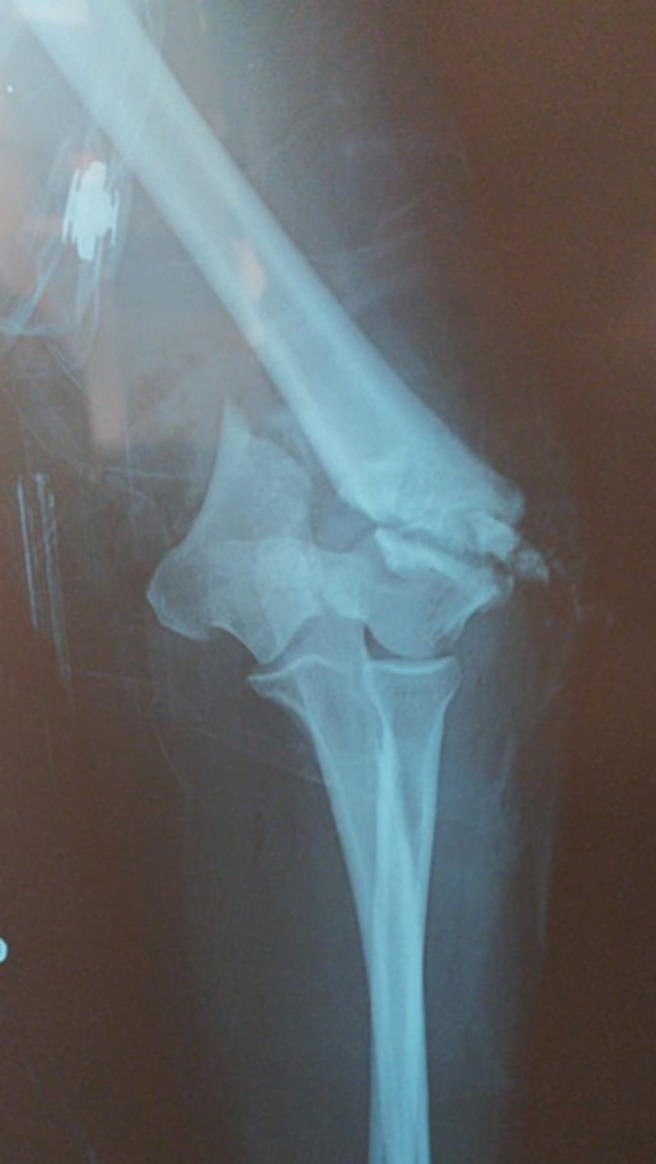
radiographie de face du coude objectivant une fracture sus et inter-condylienne de l’extrémité inférieure de l’humérus classée type C2 selon l’AO

A travers cette étude, nous avons colligé 38 cas de fractures articulaires complètes de l’EIH avec un âge moyen de 37 ans (extrêmes allant de 22-67 ans) et une nette prédominance masculine chez 71% des patients (sexe ratio H/F: 2,45). Les étiologies étaient dominées par les accidents de la voie publique (AVP) chez 78% des cas suivis par les chutes chez 22% des cas. Quarante-huit pour cent des patients étaient polytraumatisés et 20% poly-fracturés. Seuls 32% des cas étaient mono-traumatisés ne présentant qu’une fracture de l’EIH. Le côté droit a été atteint dans 70% des cas. Nous avons objectivé une ouverture cutanée punctiforme (Figure 1) chez 5% des patients, et des contusions avec large placard ecchymotique (Figure 2) chez 13% des cas. Aucune lésion vasculo-nerveuse n’a été notifiée. Un bilan radiologique standard du coude comportant deux clichés radiographiques, en deux incidences orthogonales, face stricte et profil à 90° de flexion, ont été réalisés chez tous nos patients. Treize patients (soit 34%) ont bénéficié d’un complément scanographique vu qu’ils présentaient des fractures articulaires complexes. Au terme de ce bilan radiologique et en se basant sur la classification de l’AO, la répartition des différentes fractures était comme suit: 11% étaient de type C1, 55% de type C2 (Figure 3) et 34% de type C3. Tous nos patients ont été traités chirurgicalement après un délai moyen de 7 jours (bilan préopératoire, disponibilité du matériel d’ostéosynthèse, cicatrisation des lésions cutanées). L’anesthésie générale était utilisée chez tous nos patients qui ont été installés en décubitus latéral controlatéral, sur table ordinaire, avec un garrot pneumatique placé le plus haut possible à la racine du membre à opérer.

**Figure 4:**
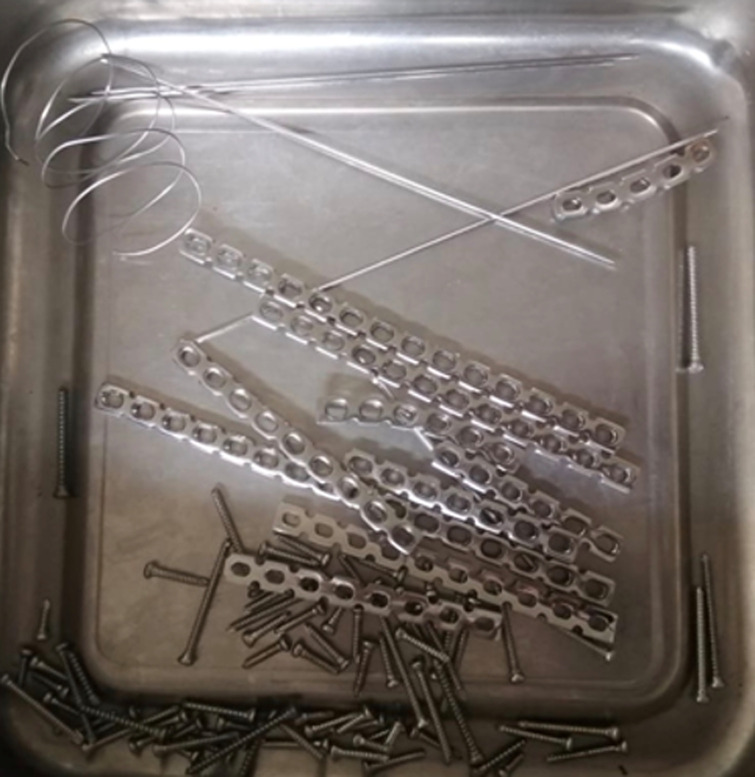
matériel d’ostéosynthèse utilisé (jeu complet de plaque de reconstruction, broche de Kirschner, fil d’acier)

**Figure 5:**
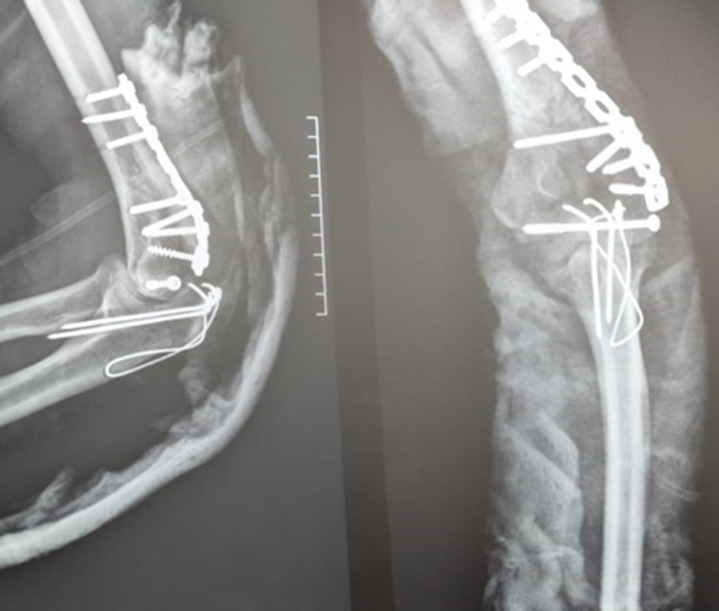
radiographie de contrôle post-opératoire d’une fracture type C1 après ostéosynthèse par une plaque postéro-latérale par voie postérieure trans-olécranienne

**Figure 6:**
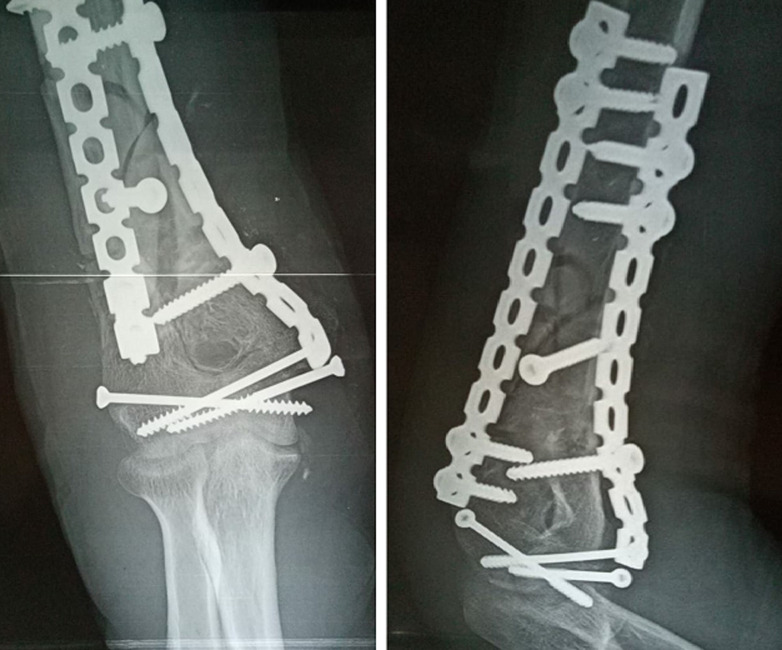
radiographie de contrôle post-opératoire montrant un montage orthogonal: une plaque postéro-latérale et une plaque médiale pour une fracture type C2

Tous les coudes opérés ont été abordés par voie postérieure trans-olécranienne. La neurolyse du nerf ulnaire a été faite chez tous les patients. Nous avons réalisé une ostéotomie intra-articulaire à la scie oscillante, 2 cm au-dessous du sommet de l’olécrane, avec un trait transversal chez 80% des cas, et une ostéotomie en chevron à apex distal chez 20%. Avant de procéder à cette ostéotomie, nous avons foré un tunnel en partant du sommet de l’olécrane et en dirigeant vers la cavité médullaire de l’ulna proximal, à l’aide d’une mèche Ø 2,7mm, afin de faciliter la réduction et l’ostéosynthèse de l’olécrane en fin d’intervention. Après ostéotomie, le fragment proximal de l’olécrane sur lequel s’insère le triceps par son tendon terminal est mis dans une compresse humide et est rabattu vers le haut afin de dégager l’EIH. Après évacuation de l’hémarthrose et extraction des petits fragments ostéo-cartilagineux libres, nous avons pu inspecter la cavité articulaire et faire le bilan lésionnel. Dans un premier temps, l’épiphyse articulaire est reconstruite, d’abord en utilisant des broches temporaires, puis elle est fixée par des vis spongieuses Ø 3,5mm, mises du capitellum vers la trochlée. Dans un deuxième temps, l’épiphyse reconstruite et synthésée est réduite sur le bloc métaphyso-diaphysaire dont les deux colonnes ont été à leur tour reconstruites et fixées en commençant par la moins comminutive, en utilisant une ou deux plaques de reconstruction (Figure 4) avec des vis spongieuses et corticales ø 3,5mm. Chez 11% des malades qui ont présenté une fracture non comminutive de la colonne latérale, nous avons utilisé une seule plaque postéro-latérale (Figure 5). Chez 89% des patients qui avaient une fracture avec comminution métaphysaire, nous avons utilisé deux plaques selon un montage dit orthogonal: une plaque postéro-latérale et une plaque médiale pour un maximum de rigidité (Figure 6). L’ostéosynthèse de l’olécrane a été assurée par embrochage-haubanage. Le nerf ulnaire a été transposé en avant chez 89% des patients. La fermeture a été faite plan par plan sur un drain de Redon aspiratif avec mise en place d’une attelle brachio-antébrachio-palmaire. Tous nos patients ont reçu une antibioprophylaxie systématique à base d’amoxicilline-acide clavulanique. En post-opératoire immédiat, nous n’avons noté aucun cas de neuropathie ulnaire. La rééducation a été entreprise précocement, dès l’atténuation des phénomènes inflammatoires (œdème, douleur): à partir de la deuxième semaine en général. Lors du suivi, nous avons noté un seul cas d’infection superficielle du site opératoire survenue à J17 post-opératoire chez un patient ayant présenté initialement un mauvais état cutané avec ecchymose et contusion. Cette infection a été jugulée par une antibiothérapie à base d’amoxicilline-acide clavulanique et des soins locaux. Nous n’avons enregistré aucun cas de sepsis profond. Sur le plan radiologique, la consolidation a été obtenue en moyenne au bout de 13 semaines (9-16 semaines) et a été retardée en cas de forte comminution chez des sujets ostéoporotiques (19% des cas). Il y’a eu aucun cas de démontage du matériel ni de pseudarthrose de l’EIH. Aucune complication de l’ostéosynthèse de l’olécrane n’a été notée dans notre série: nous avons procédé à une ablation du matériel d’ostéosynthèse (de l’olécrane et de l’EIH) chez un seul patient qui devait faire une imagerie par résonance magnétique (IRM) pour une lésion ligamentaire de son genou. Nous avons considéré comme raide le coude ayant un arc de flexion/extension inférieur à 50°, et nous avons notifié une raideur du coude chez 25% des patients. Sur le plan fonctionnel, nous avons obtenu un score de MEPS moyen de 86 avec 18% d’excellents résultats, 52% de bons résultats, 28,4% de résultats moyens et seulement 2,6% de mauvais résultats.

## Discussion

Les fractures de l’EIH représentent 2% de toutes les fractures du squelette et 33% de toutes les fractures humérales [1]. Les fractures articulaires complètes sont plus fréquentes que les fractures articulaires partielles et les fractures extra-articulaires. Sur le plan épidémiologique, bon nombre d’études rapportent une répartition bimodale de ces fractures avec une atteinte du sujet jeune de sexe masculin d’une part, et une survenue chez le sujet âgé de sexe féminin d’autre part [2]. Nos résultats semblent cadrer relativement bien avec ceux de la littérature. En effet, cette distribution pourrait être expliquée par les circonstances de survenue de ce type de fractures, notamment suite à des accidents à haute énergie (AVP) chez le jeune s’inscrivant le plus souvent dans un cadre de polytraumatisme, ou suite à une chute domestique banale chez des femmes ménopausées ostéoporotiques. Quant à l’examen clinique, il permet de rechercher les détresses vitales en cas de polytraumatisme, ainsi que les lésions associées osseuses, cutanées ou vasculo-nerveuses. De surcroit, le bilan radiologique standard permet de confirmer le diagnostic et de classer la fracture. Cependant, Hua *et al*. [3] et Nolan *et al*. [4], recommandent la réalisation systématique de la tomodensitométrie (TDM) du coude, en complément du bilan standard initial, pour toute fracture articulaire complète, et ce pour une meilleure caractérisation lésionnelle et donc une meilleure planification pré-opératoire (voie d’abord et montage). Dans notre série, la TDM n’a été faite que chez 10 patients (soit 26%), qui ont présenté une fracture complexe classée C3 selon l’AO.

Concernant le traitement de ces fractures complexes, il est essentiellement chirurgical par ostéosynthèse. Dans certains cas bien précis, liés au terrain (demande fonctionnelle réduite, tares multiples, risque anesthésique majeur) et/ou à la fracture (forte comminution ou véritable fracas du coude), le traitement orthopédique pourrait être indiqué [5]. Par ailleurs, certaines études se sont intéressées à la place de l’arthroplastie du coude dans l’arsenal thérapeutique de ce type de fractures. Ainsi Barci, dans son étude comparative entre le traitement conservateur et le traitement prothétique, et vu les bons résultats qu’il a obtenus après un recul de 10 ans (bonne récupération fonctionnelle, longévité de la prothèse), a conclu que l’arthroplastie devrait être indiquée en première intention en cas de fracture type C3 chez des patients âgés [6]. Toutefois, et malgré que l’ostéosynthèse reste le traitement le plus souvent indiqué pour ces fractures, les publications s’intéressant aux voies d’abord ainsi qu’à la technique chirurgicale ne sont pas unanimes et la majorité d’entre elles comparent les différentes voies postérieures qui sont les plus utilisées [7]. Ces dernières sont classées, en fonction de leur attitude vis-à-vis de l’appareil extenseur (triceps, olécrane) en voies para-tricipitales, trans-tricipitales et trans-olécraniennes. D’après Azboy, la voie transcipitale est supérieure à la voie trans-olécranienne, puisqu’elle évite les complications liées à cette dernière et qui sont: le risque de pseudarthrose ou de retard de consolidation de l’olécrane, la formation d’ossifications hétérotopiques, la saillie des broches sous la peau, la nécessité de réintervention pour ablation du matériel) [8]. Néanmoins, Wilkinson et Stanley ont démontré que le jour obtenu sur la surface articulaire est d’autant meilleur que l’on s’affranchit de l’obstacle olécranien. Ainsi, les voies paratricipitales, trans-tricipitales et trans-olécraniennes, exposent-elles respectivement 35%, 46% et 57% de la surface articulaire [9]. Ces résultats vont dans le même sens que ceux notifiés par la méta-analyse publiée par Chen et qui a conclu que la voie trans-olécranienne est la meilleure voie d’abord en cas de fracture articulaire complète de l’EIH puisqu’elle permet une meilleure exposition articulaire nécessaire pour une réduction anatomique parfaite, seule garante d’une bonne récupération fonctionnelle ultérieure. Les complications liées à cette voie peuvent être évitées grâce à une technique d’embrochage-haubanage rigoureuse [10]. Quelle que soit la voie d’abord utilisée, l’ostéosynthèse de ces fractures répond à des critères bien précis: la réduction doit être anatomique et parfaite; le matériel doit être configuré selon un montage solide pour pouvoir autoriser une rééducation précoce. L’ostéosynthèse des fractures articulaires complètes de l’EIH comprend deux étapes: la reconstruction de l’épiphyse (par vissage) et sa solidarisation à la diaphyse par une ou deux plaques (LECESTRE, tiers de tube, anatomique pré-moulée, verrouillée, mini-invasive ou de reconstruction). Généralement, deux types de configuration sont utilisés: montage par deux plaques parallèles (médiale et latérale) ou orthogonales (médiale et postéro-latérale) et les résultats sont similaires comme l’a démontré la méta-analyse de Yu *et al*. [11]. Toutefois, Shih *et al*. recommande le montage par deux plaques parallèles pour un maximum de stabilité [12]. La raideur du coude est la complication la plus fréquente des fractures articulaires de l’EIH puisqu’elle est notifiée chez à peu près 33% des patients par les séries de la littérature [13]. Dans le présent travail, nous rapportons un taux de raideur moindre. La pseudarthrose, quant à elle, complique 2-10% des fractures selon Helfet *et al*. [14]. Elle est essentiellement due à un montage par une seule plaque puisqu’il a été démontré par l’étude de Theivendran que l’utilisation de 2 plaques permet d’obtenir une consolidation dans 89 à 100% [15]. A côté du montage insuffisant, la forte comminution et l’ostéoporose favorisent également la pseudarthrose. Ces mêmes facteurs de risque exposent au démontage du matériel dont le taux varie de 7 à 27% [16]. Dans notre série, nous n’avons notifié aucun cas de pseudarthrose ni de démontage.

Pour ce qui est des complications septiques, Athwal *et al*. [17] et Lawrence *et al*. [18] ont rapporté respectivement un taux d’infection de 12% et de 15,7%, taux supérieurs à celui notifié par notre série. Ces complications sont essentiellement favorisées par la présence de lésions cutanées. L’atteinte du nerf ulnaire peut être causée par la fracture elle-même et apparaitre en pré-opératoire, comme elle peut être due à la manipulation chirurgicale ou à l’irritation par le matériel d’ostéosynthèse. Les taux varient selon les séries et peuvent atteindre 51% des cas [19]. Il a été récemment démontré que la transposition antérieure du nerf ulnaire ne diminue pas le risque d’atteinte nerveuse, au contraire, certains auteurs ont même prouvé qu’elle l’augmenterait [20]. Les séries de la littérature ainsi que la nôtre confirment les bons résultats fonctionnels du traitement chirurgical des fractures articulaires complexes de l’EIH. Le MEPS moyen varie entre 82 et 94,17 selon les études [21, 22].

## Conclusion

Les résultats cliniques et radiologiques de notre étude étaient très encourageants, la consolidation a été survenue dans la plupart des cas dans un délai raisonnable, et le taux de complication était très relativement faible. La voie postérieure trans-olécranienne semble être la meilleure voie d’abord puisqu’elle permet une bonne exposition articulaire, condition sine qua non pour une restitution anatomique parfaite et une ostéosynthèse stable afin d’entreprendre une rééducation précoce et adaptée, seule garante d’une bonne récupération fonctionnelle.

### Etat des connaissances sur le sujet

- Les fractures sus et inter-condyliennes de l’humérus distal sont des lésions articulaires relativement rares et réputées graves;
- Complications assez fréquentes;
- Le choix de la voie d’abord reste controversé.

### Contribution de notre étude à la connaissance

- Notre étude confirme les données épidémiologiques et anatomopatholo giques décrites dans la littérature;
- La voie d’abord postérieure trans-olécranienne du coude semble être la meilleure voie pour traiter les fractures sus et inter-condyliennes de l’humérus distal de l’adulte;
- Elle permet une bonne exposition articulaire pour une meilleure réduction fracturaire et une fixation par un montage stable et solide pour une rééducation précoce et adaptée.

## Remerciements

Nous remercions l’ensemble des patients et le personnel du service de Chirurgie Orthopédique et Traumatologique de l’hôpital Ibn Tofail (CHU Mohammed VI-Marrakech) qui ont rendu possible cette étude.
