# Corrigendum: Meryem Lemsanni *et al*. Fractures sus et inter-condyliennes de l’humérus distal chez l’adulte doi: 10.11604/pamj.2020.36.346.24516

**DOI:** 10.11604/pamj.2021.38.152.28075

**Published:** 2021-02-10

**Authors:** Meryem Lemsanni, Rachid Chafik, Mohamed Madhar, Hanane Elhaoury, Youssef Najeb

**Affiliations:** 1 Service de Chirurgie Orthopédique et Traumatologique, Hôpital Ibn Tofail, Centre Hospitalier Universitaire Mohammed VI, BP 40000, Marrakech, Maroc

**Keywords:** Corrigendum, fracture, humérus distal, articulaire, sus et inter-condylienne, ostéosynthèse, abord postérieur, trans-olécranien, Corrigendum, fracture, distal humerus, articular, sub and intercondylian, osteosynthesis, posterior approach, transolecranon

## Abstract

Ce corrigendum modifie l´article “Fractures sus et inter-condyliennes de l´humérus distal chez l´adulte”. Access corrected manuscript Pan African Medical Journal. 2020; 36: 346. doi: 10.11604/pamj.2020.36.346.24516.

## Abstract (en)

This corrigendum modifies the original article: “Sub- and intercondylar fractures of the distal humerus in adults”. Access corrected manuscript Pan African Medical Journal. 2020; 36: 346. doi: 10.11604/pamj.2020.36.346.24516.

## Corrigendum

Dans la version originale de l´article, une erreur a eu lieu par inadvertance [1]. En effet, il s´agit d´une erreur concernant la Figure 6 qui montre un montage orthogonal utilisé pour l´ostéosynthèse d´une fracture de l´humérus distal (type C2 selon la classification de l´AO) par un abord trans-tricipital. Or, notre série a inclus uniquement les fractures traitées par voie postérieure trans-olécranienne. Nous avons joint la version corrigée de cette figure (Figure 1) qui montre un montage orthogonal pour une fracture de l´humérus distal opérée par voie postérieure trans-olécranienne et correspondant ainsi à la technique chirurgicale utilisée chez tous les patients de notre série. Cette erreur ne modifie en aucun cas le contenu ni la conclusion scientifique de notre article.

**Figure 1:**
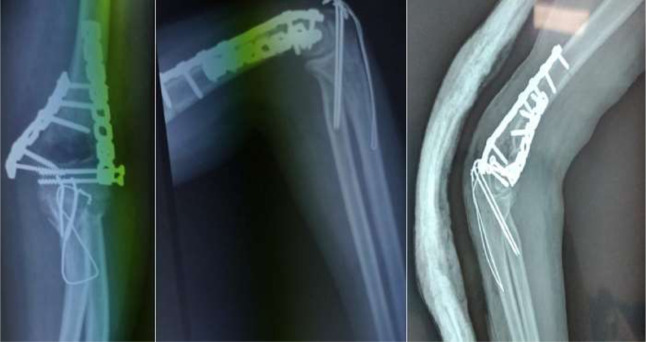
radiographie de contrôle post-opératoire montrant un montage orthogonal: une plaque postéro-latérale et une plaque médiale pour une fracture type C2
